# Bridging the gap: methodological challenges and innovations in systematic reviews of machine learning models in healthcare

**DOI:** 10.3389/frai.2026.1739192

**Published:** 2026-05-08

**Authors:** Brenda Mbouamba Yankam

**Affiliations:** Faculty of Mathematics, Ruhr University Bochum, Bochum, Germany

**Keywords:** healthcare, machine learning, meta-analyses, methodological challenges, prediction models, systematic reviews

## Highlights

- Machine learning prediction models differ fundamentally from static medical interventions due to their data dependence, retraining, and temporal instability.
- Current systematic review methodologies are poorly suited to ML evidence, contributing to optimism bias, limited external validation, and non-comparable performance estimates.
- Narrative synthesis is preferable when models lack external validation, while meta-analysis or network meta-analysis should be reserved for transparently reported and externally validated models.
- AI-specific tools and frameworks, such as PROBAST+AI, TRIPOD+AI, and PRISMA-AI, are essential for assessing bias, transparency, and applicability in ML studies.
- External validation, fairness assessment, and reproducibility remain critical gaps in the ML literature and should be prioritized in both primary studies and evidence synthesis.
- Ongoing SRs, supported by automation and active learning, offer a practical path forward for synthesizing rapidly evolving ML evidence.

## 1 Introduction

Machine learning algorithms have proliferated across the healthcare sector, with applications ranging from sepsis prediction and cancer diagnosis to surgical outcome forecasting and personalized treatment selection (Sendak et al., 2020; Nagendran et al., 2020; Rajkomar et al., 2019). Over 10,000 ML algorithms have been described in published SRs, with exponential growth over the past 5 years (Kolasa et al., 2024). This rapid expansion has outpaced the development of rigorous methodological frameworks. ML models differ fundamentally from traditional medical interventions in that they are data-dependent, adaptive, and subject to temporal performance drift. As data and populations evolve, ML models' behavior can shift, making it difficult to assess them using traditional research methods (Finlayson et al., 2021).

Despite thousands of published models, few have been widely implemented. The diabetic retinopathy screening algorithms decreased when applied to populations other than the training data (Beede et al., 2020). These failures highlight the importance of reliable evidence synthesis in correctly describing real-world performance, identifying bias, and guiding implementation.

Also, a study by (Kolasa et al. 2024) of ML in healthcare revealed substantial methodological inadequacies: 44% lacked reported accuracy metrics, 72% omitted sensitivity data, 75% omitted specificity, only 53% reported internal validation, fewer than 1% underwent external validation, and risk of bias assessment was frequently absent. Among these deficiencies, the near absence of external validation is the most critical barrier, as it fundamentally limits the ability to assess real-world model performance and to determine appropriate synthesis methods. These gaps arise from both poor primary study reporting and fundamental challenges in adapting SR approaches to ML evidence (Liu et al., 2020; Norgeot et al., 2020).

While Kolasa et al. (2024) review systematic literature reviews to characterize the availability, performance reporting, and adoption-related challenges of AI solutions in healthcare, the present work addresses a different methodological question. Specifically, it focuses on how SRs of machine learning prediction models should be designed and conducted, given issues such as limited external validation, algorithmic bias, and model instability. This review offers methodological guidance for evidence synthesis, rather than an assessment of AI availability or adoption readiness, through a decision framework and the operationalization of emerging AI-specific bias and reporting tools.

Unlike prior reviews that primarily summarize developments in AI in healthcare, this study adopts a critical and analytical perspective. It systematically evaluates methodological strengths, limitations, and inconsistencies across existing systematic reviews of ML prediction models. By synthesizing empirical evidence and comparing methodological approaches, the review identifies areas of consensus, highlights persistent deficiencies, and proposes solutions to improve the reliability and transparency of evidence synthesis. It highlights how SRs should be conducted to adequately test ML prediction models. Specifically, it documents methodological challenges in ML SRs, critically evaluates emerging solutions, proposes a decision framework linking model characteristics to appropriate SR methods, and outlines a research agenda prioritizing external validation, fairness assessment, and ongoing reviews.

## 2 Background: why machine learning evidence synthesis is different

ML prediction models differ fundamentally from medical interventions, creating unique challenges for SR. Unlike drugs or procedures, ML models are dynamic and can be retrained continuously, so the version evaluated in a study may differ from the deployed version (Finlayson et al., 2021). Model performance is data-dependent: a model trained on European populations may fail when applied elsewhere (Obermeyer et al., 2019). The opacity of deep learning models complicates evaluation, as reviewers cannot easily assess learned patterns for clinical plausibility (Rudin, 2019). Implementation variability, from electronic health record (EHR) differences to preprocessing pipelines, further affects performance (Sendak et al., 2020), and inconsistent reporting of metrics hinders comparison (Wynants et al., 2020).

In addition, real-world retraining further distinguishes ML models from static medical interventions. A prominent example is the epic sepsis model (ESM) (Wong et al., 2021) a proprietary ML algorithm deployed across hundreds of hospitals in the United States. Independent evaluations demonstrated substantial performance degradation when the model was applied to new clinical settings, with discrimination approaching random performance despite high accuracy reported during development (Wong et al., 2021).

These findings were attributed to dataset shift, differences in clinical workflows, and evolving coding and treatment practices. In response, the model was recalibrated and retrained using updated EHR data to improve local performance. As a result, the ESM deployed at different sites or time points represents distinct algorithmic versions with materially different behavior. Unlike pharmaceuticals or procedures, whose composition and mechanism remain fixed after approval, ML models are mutable, data-dependent, and temporally unstable, which complicates systematic review and meta-analysis unless model versioning and retraining histories are explicitly accounted for.

Despite these challenges, systematic reviews (SRs) remain essential for the comparative evaluation of algorithms, identification of hidden biases, and establishment of performance benchmarks. Network meta-analyses (NMAs) have identified leading sepsis prediction models, demonstrating that DeepSOFA and convolutional neural networks outperform traditional approaches (Sendak et al., 2020). Meta-analyses have also quantified the “optimism gap” between internal and external validation, which can reach 0.20 in studies with suboptimal design (Wynants et al., 2020).

Artificial intelligence tools are increasingly integrated into the SR process. Active-learning platforms such as ASReview (an active learning software for systematic reviews that uses ML to prioritize relevant studies during screening) and Rayyan, a web-based systematic review management platform, reduce the screening workload (van de Schoot et al., 2021), while large language models (LLMs) assist with data extraction under human supervision (Daraio et al., 2026). Topic modeling further supports the identification of patterns across extensive literature collections (Atkinson, 2024). Although these tools enhance efficiency, they introduce new challenges related to reproducibility and version control.

## 3 Methodological challenges and pitfalls

This work is a narrative methodological review that critically evaluates published SRs of ML models in healthcare, rather than a formal SR or meta-analysis of primary studies.

To identify relevant literature informing this methodological review, a structured exploratory search was conducted in major bibliographic databases, including PubMed, Web of Science, Scopus, and Google Scholar. Searches focused on publications addressing systematic reviews, meta-analyses, and methodological evaluations of machine learning models in healthcare. Search terms included combinations of “machine learning,” “artificial intelligence,” “systematic review,” “meta-analysis,” “prediction models,” and “healthcare.” Priority was given to methodological studies, systematic reviews of ML prediction models, and publications discussing bias, reporting standards, and validation practices in clinical ML research.

Studies were considered eligible if they (1) evaluated or discussed methodological aspects of systematic reviews or meta-analyses of machine learning prediction models in healthcare; (2) provided empirical analyses of reporting quality, bias assessment, validation practices, or reproducibility in clinical ML research; (3) introduced, evaluated, or compared methodological frameworks, reporting guidelines, or bias assessment tools relevant to ML evidence synthesis such as PROBAST + AI, TRIPOD + AI, PRISMA-AI. Publications in English were prioritized; however, no language restriction was formally applied. Editorials, purely technical algorithm development papers without clinical context, and studies unrelated to evidence synthesis were excluded. The aim of this research was not to produce an exhaustive systematic review but to identify representative literature illustrating the methodological challenges and emerging solutions relevant to evidence synthesis for ML models.

Systematic reviews of ML models encounter multiple methodological challenges, including heterogeneity, incomplete reporting, bias, limited reproducibility, and insufficient external validation. Studies often assess a wide range of algorithms, such as logistic regression, random forests, and deep neural networks, using diverse data types and outcomes, which hinders the pooling of results (Christodoulou et al., 2019). Network meta-analysis can partially address algorithmic heterogeneity but relies on assumptions that are frequently violated in ML research (Sendak et al., 2020).

The heterogeneity of ML models used in healthcare research operates across at least three distinct dimensions, each with specific and compounding consequences for evidence synthesis. The first is structural heterogeneity, referring to fundamental differences in model architecture and learning paradigm. (Christodoulou et al. 2019) conducted a systematic review of 71 studies comparing logistic regression and machine learning for clinical prediction tasks, identifying 282 head-to-head comparisons, and found no evidence of superior discriminative performance of ML over logistic regression at low risk of bias, with a median AUC difference of 0.00 (95% CI, −0.18 to 0.18); critically, calibration was not reported in 79% of included studies, and 68% showed potential bias in validation procedures. This finding is pivotal for evidence synthesis: it demonstrates that the choice of model family does not reliably predict performance advantage, yet structural differences between model classes do determine interpretability, calibration behavior, and failure modes in ways that AUC alone cannot capture. Rudin (2019) argued compellingly that deep neural networks and other black-box models used in high-stakes healthcare decisions create a fundamental problem of opacity, where even *post-hoc* explanation methods do not provide clinically meaningful insight into how predictions are generated, and advocated instead for inherently interpretable models that can be directly scrutinized without separate explanation tools. For systematic reviewers, this structural heterogeneity means pooling AUC estimates across logistic regression, random forests, and deep neural networks is methodologically analogous to pooling pharmacologically distinct drug classes: the summary estimate reflects no single clinically meaningful entity, and the constituent models differ not only in accuracy but in their transparency, calibration behavior, data requirements, and the conditions under which they are expected to generalize. Nagendran et al. (2020) found that most studies were at high risk of bias, only nine were prospective, and fewer than 5% were tested in a real-world clinical setting, with adherence to TRIPOD reporting standards being suboptimal in over 40% of items; these findings reflect the same structural heterogeneity problem, as studies using convolutional neural networks could not be meaningfully compared with studies using simpler classification approaches. The second dimension is data heterogeneity. ML model performance is inseparable from the characteristics of the data used for training and validation: source population, sample size, class balance, data modality such as structured EHR, imaging, natural language processing, coding conventions, and temporal period all materially affect the AUC and calibration values that a model produces. Wynants et al. (2020) demonstrated that performance commonly declined substantially when models were applied to populations other than the one used for development, with the optimism gap between internal and external validation reaching 0.20 in studies with suboptimal design; this gradient of performance degradation is driven at least partly by data heterogeneity rather than algorithmic failure, yet this distinction is obscured when internally and externally validated studies are pooled without stratification. When studies with heterogeneous training data are pooled without such stratification, *I*^2^ statistics will be high, and the pooled estimate conflates algorithmic differences with data-driven variance, producing a misleading summary. The third dimension is outcome and task heterogeneity. Gao et al. (2024) were able to conduct comparative synthesis only after restricting inclusion to studies sharing sufficiently similar outcome definitions and populations, illustrating that outcome operationalization differences, such as using Sepsis-1 vs. Sepsis-2 vs. Sepsis-3 definitions, or predicting onset at different time horizons, alter base rates, sensitivity-specificity trade-offs, and the clinical interpretation of the AUC in ways that make cross-study metric comparisons unreliable without explicit harmonization. Pre-specified subgroup analyses stratifying by model family, data modality, and outcome definition should therefore be regarded as mandatory rather than exploratory in ML systematic reviews, and the transitivity assumptions underlying network meta-analysis require explicit justification and sensitivity testing before being applied to this heterogeneous body of evidence.

Reporting practices remain inconsistent. A review by Kolasa et al. (2024) identified that accuracy was reported in 56% of studies, sensitivity in 28%, specificity in 25%, calibration in fewer than 10%, and confidence intervals in fewer than 40%. Many studies present best-case performance results, leading to an overestimation of effectiveness (Wallach et al., 2018). Meta-analyses of COVID-19 diagnostic models showed that complete diagnostic tables were available in only nine of 62 studies (Roberts et al., 2021), showing the fragility of current evidence synthesis.

Moreover, bias assessment represents another significant limitation. Data leakage, where training data contaminates validation sets, affects up to 40% of imaging studies (Cacciamani et al., 2023). Algorithmic bias, such as systematic underestimation of risk in Black patients, remains infrequently reported (Obermeyer et al., 2019). The Prediction model Risk Of Bias ASsessment Tool + Artificial Intelligence (PROBAST + AI) framework addresses ML-specific risks, including fairness, model interpretability, and model updating (Moons et al., 2025), but uptake remains limited due to its complexity and resource demands.

Reproducibility is constrained, with only 19% of ML studies sharing code, 8% sharing model weights, and fewer than 2% sharing training data (Gundersen et al., 2018). Continuous model updates further complicate ongoing evidence synthesis. External validation remains rare, reported in fewer than 1% of studies, yet is essential, as performance commonly declines from 0.85 to 0.72 in c-statistics when evaluated on independent datasets (Kolasa et al., 2024; Ramspek et al., 2021). Addressing these methodological weaknesses is critical for establishing reliable and trustworthy ML evidence synthesis.

Two recent systematic reviews provide direct empirical grounding for these methodological concerns. Alhumaidi et al. (2025) found that the most widely deployed ML methods, random forest (42% of studies), logistic regression (37%), and support vector machines (32%), were applied predominantly to cardiovascular disease (33%), cancer (16%), and neurological disorders (11%), with real-world evidence sourced almost entirely from EHRs, patient registries, and wearable devices. Critically, 60% of included studies encountered challenges related to data quality, model interpretability, and ensuring generalizability across diverse patient populations. Alhumaidi et al. (2025) specifically documented that ML models trained on real-world data frequently inherit biases stemming from demographic imbalances or regional healthcare differences, and that if left unaddressed, these biases produce healthcare disparities whereby ML-driven decisions inaccurately represent racial and ethnic minority populations or specific patient groups, providing direct empirical evidence that algorithmic bias is not a theoretical risk but a documented feature of currently deployed systems. They further found that the opaque decision-making processes of deep neural networks constrain clinical trust and verification, even when high accuracy is reported, underscoring the real-world consequences of the interpretability gap for evidence synthesis. Rakers et al. (2024) identified 43 predictive ML algorithms implemented in primary care, of which 25 were commercially available and CE-marked or FDA-approved. Assessing evidence availability across five phases of the AI life cycle, from preparation through impact assessment, against the Dutch Artificial Intelligence Predictive Algorithm guideline, they found that evidence was most frequently reported only for the development phase, with phase 1 (preparation) and phase 5 (impact assessment) showing the lowest evidence availability at 19% and 30%, respectively. Overall, only 12 of 43 algorithms (28%) achieved approximately half of their maximum individual evidence availability score, and algorithms from peer-reviewed literature showed substantially higher evidence availability than those from FDA-approved or CE-marked databases (45% vs. 29%). Rakers et al. (2024) concluded that this gap reflects an urgent need for transparent and consistent reporting of quality criteria across the full algorithm life cycle. These findings concretely illustrate why the challenges of algorithmic bias, data quality limitations, and insufficient external validation are not merely conceptual: they are consistently empirically documented across disease domains, data types, and regulatory settings, and they directly impair the ability of systematic reviewers to synthesize ML evidence reliably.

## 4 Emerging solutions and innovations

The discussion of active learning systems and LLMs in this review is based on previously published evaluations of these tools in SR workflows; no AI-assisted review tools were directly implemented or empirically assessed as part of the present study.

Emerging solutions address bias, transparency, and heterogeneity in ML systematic reviews. Reporting and bias assessment frameworks, PROBAST + AI (Moons et al., 2025), TRIPOD + AI (Collins et al., 2024), and Preferred Reporting Items for Systematic Reviews and Meta-Analyses (PRISMA-AI) (Page et al., 2021), provide structured guidance for both primary studies and SRs. Network meta-analysis enables comparisons across heterogeneous algorithms, adjusting for study-level covariates such as data type or feature engineering (Gao et al., 2024). While these frameworks represent substantial methodological progress, their practical implementation remains uneven across the literature. Variability in adherence, limited reviewer expertise, and inconsistent reporting in primary studies restrict their full potential. Consequently, systematic reviewers must not only apply these tools but also critically evaluate their suitability, limitations, and contextual applicability.

However, each of these tools has significant drawbacks that call for careful consideration. PROBAST + AI, developed by Moons et al. (2025), is the most comprehensive bias assessment tool currently available for ML prediction models. It comprises four domains: participants, predictors, outcome, and analysis, each with multiple signaling questions requiring granular methodological judgement about data preprocessing pipelines, feature selection procedures, and model updating protocols. In practice, this complexity contributes to the low uptake observed in published SRs, as Moons et al. (2025) themselves acknowledged, and introduces inter-rater variability when reviewers lack statistical or ML expertise. A further limitation not addressed in the current version is the absence of validated rules for aggregating domain-level risk-of-bias judgements into an overall study-level rating, leaving room for subjective interpretation that undermines cross-study comparability. The tool also offers limited guidance on assessing bias in continuously retrained or federated models, where the distinction between development and validation data may be difficult to ascertain from published reports. TRIPOD + AI, updated by Collins et al. (2024), similarly represents a substantive advance in reporting guidance for ML prediction model studies. However, its items remain oriented primarily toward single-point-in-time model development and validation. As Collins et al. (2024) note, the statement was designed to cover a broad range of ML methods and therefore cannot prescribe model-specific reporting elements in depth; this means that critical details such as hyperparameter tuning protocols, ensemble composition, and training data versioning, all of which materially affect reproducibility and performance interpretation, may still go unreported even in TRIPOD + AI-compliant manuscripts. Adherence to TRIPOD + AI alone does not guarantee that a model is free of data leakage or that reported performance estimates are generalizable, as it is a reporting standard rather than a quality filter. Cacciamani et al. (2023) introduced the PRISMA – AI extension for ML-focused systematic reviews, but its utility is constrained by the very reporting deficiencies it seeks to remedy: if primary studies do not report the items required by PRISMA – AI, such as calibration statistics, fairness metrics, or code availability, systematic reviewers cannot extract or synthesize them even with the best intentions. These frameworks are necessary but not sufficient conditions for rigorous ML evidence synthesis, and their adoption should be accompanied by structured training, inter-rater reliability testing, and continued empirical evaluation of their impact on review reproducibility and completeness.

AI-assisted automation streamlines SR processes. Active learning and LLMs can reduce screening time while maintaining accuracy (van de Schoot et al., 2021; Daraio et al., 2026). Ongoing systematic reviews integrate automated literature monitoring to maintain currency in rapidly evolving ML fields (Elliott et al., 2014).

In parallel with methodological and automation advances, recent initiatives demonstrate tangible progress in open data and code sharing for medical ML. Recent studies indicate progress in open science for clinical machine learning. Examples include the MultiCaRe multimodal dataset with shared tools (Offidani et al., 2025), publicly cataloged ultrasound datasets with open-source models (Alsharid et al., 2025), and large multimodal resources such as UniMed (Khattak et al., 2024), collectively supporting improved reproducibility, benchmarking, and generalizability.

## 5 Decision framework for conducting machine learning systematic reviews

While recent initiatives such as PROBAST + AI, TRIPOD + AI, and PRISMA – AI have substantially advanced reporting and bias assessment in ML studies, they do not specify how to select evidence synthesis methods based on model validation status. Existing SRs frequently apply meta-analysis or comparative synthesis without distinguishing between internally validated models and those evaluated on independent external datasets. The decision framework proposed here addresses this gap by explicitly linking validation status to permissible review methods: narrative synthesis for internally validated models, meta-analysis for externally validated models, and network meta-analysis when multiple externally validated algorithms are compared. Integrating AI-specific bias and reporting tools into a validation-driven synthesis strategy, this approach operationalizes emerging guidelines and provides methodological guidance that is currently absent from the SR literature.

**Figure 1:**
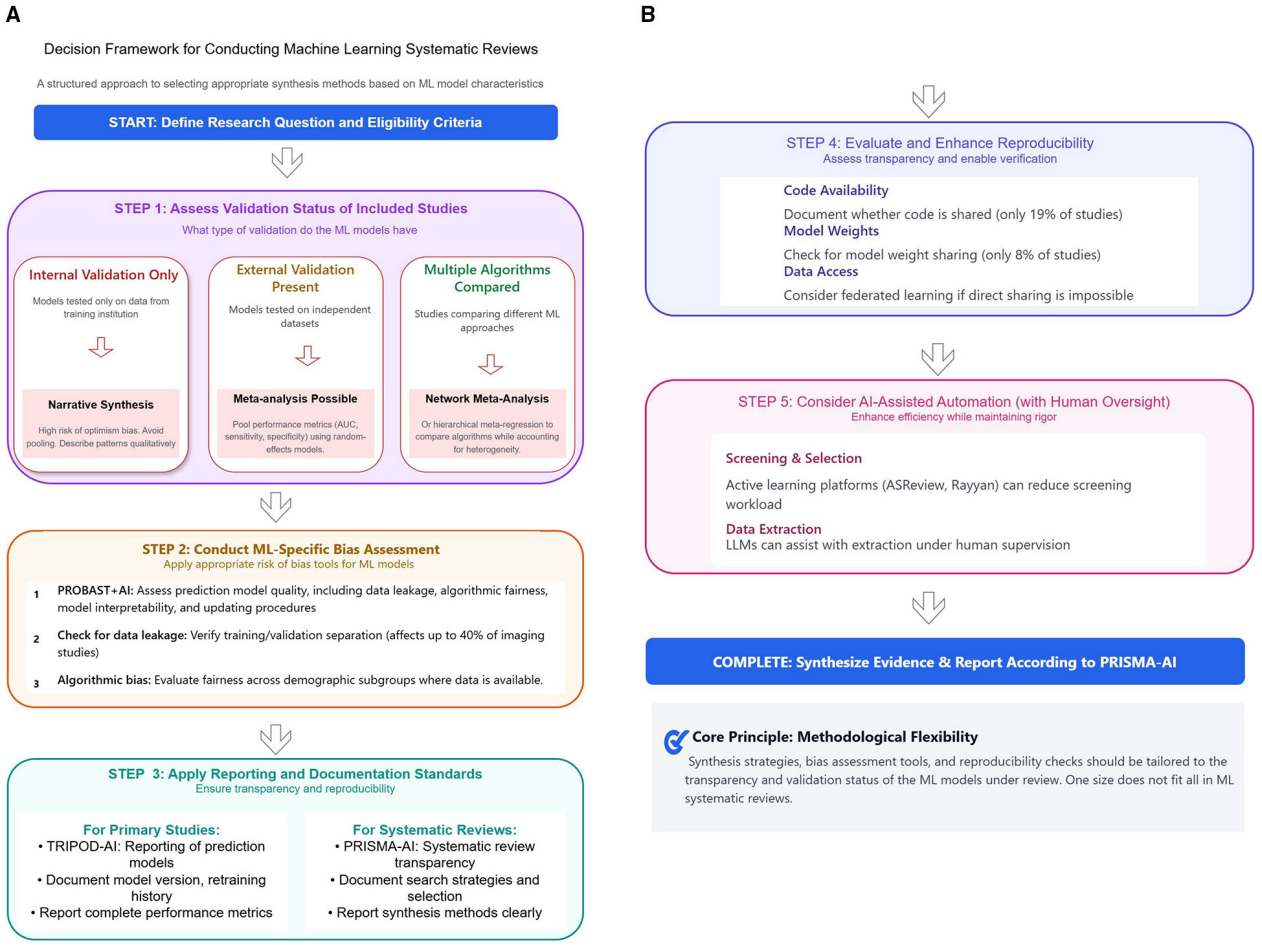
**(A, B)** Decision framework for conducting machine learning systematic reviews.

Systematic reviews of ML models require methodological approaches tailored to evidence characteristics, particularly validation status (Figures 1A, B). When studies rely solely on internal validation, narrative synthesis is preferred due to optimism bias, as internally validated models systematically overestimate performance (Wynants et al., 2020). Externally validated models permit meta-analysis of performance metrics such as area under the curve, sensitivity, and specificity using random-effects models. When multiple algorithms are compared, network meta-analysis or hierarchical meta-regression enables comparative evaluation while accounting for heterogeneity (Gao et al., 2024; Debray et al., 2013). This validation-driven approach ensures synthesis methods align with the reliability and generalizability of the underlying evidence.

To operationalize this validation-driven approach, reviewers should follow a sequential five-step decision process grounded in the existing evidence base, corresponding to the workflow depicted in Figures 1A, B.

Step 1: assess validation status and select synthesis method is the gateway decision. The reviewer determines the proportion of included studies that report external validation on an independent dataset. The near-complete absence of external validation in the ML literature, reported by Kolasa et al. (2024) in fewer than 1% of reviewed ML studies, means that in most current ML systematic reviews, narrative synthesis will be the only methodologically defensible option. External validation is essential not merely as a regulatory or quality assurance step but because model performance on development data is an unreliable proxy for real-world performance due to overfitting, case-mix differences, and temporal drift; internal validation estimates therefore carry inherent optimism bias that makes quantitative pooling misleading (Ramspek et al., 2021). When fewer than 25% of included studies have undergone external validation, narrative synthesis is mandatory: it should describe the range of reported metrics such as AUC, sensitivity, specificity, calibration slope, characterize algorithmic and data heterogeneity, and explicitly flag the optimism bias risk documented by Wynants et al., who found that performance commonly declined by as much as 0.20 in AUC between internal development and external evaluation in COVID-19 prediction model studies. The reviewer must also document all reported performance metrics and flag any reported only under internal validation as subject to optimism bias before proceeding to subsequent steps. When 25% or more of included studies are externally validated and report a common performance metric with accompanying precision estimates, bivariate or univariate random-effects meta-analysis is appropriate (the meta-analysis pathway). Pooling should be restricted to studies sharing the same outcome, population type, and validation design, consistent with the individual participant data framework described by Debray et al. (2013), who demonstrated that ignoring study-level differences in case mix and validation design substantially inflates heterogeneity estimates and biases pooled performance summaries. Heterogeneity should be quantified using *I*^2^ and explored through pre-specified subgroup analyses stratified by data type, model family, or population, as applied by Wynants et al. (2020) in their COVID-19 prediction model review. When two or more distinct algorithm types are each represented by at least three externally validated studies, network meta-analysis or hierarchical meta-regression is warranted to enable indirect comparisons across algorithms while adjusting for study-level covariates (the NMA pathway). Gao et al. (2024) demonstrated the feasibility of this approach in their systematic review and network meta-analysis of ML algorithms for sepsis prediction, identifying DeepSOFA and convolutional neural networks as superior to conventional scoring systems, but only by restricting the evidence base to studies with comparable outcome definitions, population characteristics, and validation designs. Their experience illustrates both the potential and the stringent data quality requirements of NMA in this context.

Step 2: conduct ML-specific bias assessment applies in parallel with and prior to all three synthesis pathways. This involves applying PROBAST + AI as described by Moons et al. (2025) to each included study across all four domains, participants, predictors, outcome, and analysis, before any synthesis takes place. Studies rated as high overall risk of bias should be excluded from quantitative pooling and retained only in sensitivity analyses. Reviewers should use PROBAST + AI's analysis domain to flag data leakage, improper handling of missing data, and inadequate sample sizes for the number of predictors, each of which inflates apparent model performance. Algorithmic bias should additionally be assessed by examining whether model performance is reported stratified by demographic subgroup such as age, sex, race, or socioeconomic status, as demonstrated necessary by Obermeyer et al. (2019) and corroborated empirically by Alhumaidi et al. (2025).

Step 3: Apply AI-specific reporting standards requires that data extraction and reporting of review findings be conducted against the TRIPOD + AI checklist for primary studies and the PRISMA – AI checklist as introduced by Cacciamani et al. (2023) for the systematic review itself. Reviewers should document which TRIPOD + AI items were met by each included study, as this quantifies the degree of completeness of reporting and determines how many studies can contribute to each synthesis pathway. Gaps in compliance should be reported transparently, following the precedent of Nagendran et al. and Rakers et al., who each found that adherence to reporting standards was suboptimal across the majority of included algorithms.

Step 4: evaluate reproducibility requires the reviewer to document, for each included study, whether model code, trained weights, and training data are publicly available. As established by Gundersen et al. (2018), fewer than 20% of ML studies in healthcare provide sufficient artfacts for independent reproduction. Reproducibility assessment should be reported as a structured summary alongside the risk-of-bias table, and studies with no shared artfacts should be flagged as having limited verifiability. Where direct data sharing is not possible, the reviewer should note whether federated learning or privacy-preserving validation approaches were used, as these represent emerging mechanisms for achieving external validation without data transfer.

Step 5: consider AI-assisted automation with human oversight applies to the conduct of the systematic review process itself rather than to the ML models under review. Reviewers should consider active learning tools such as ASReview, as described by van de Schoot et al. (2021), or large language model-assisted screening as evaluated by Daraio et al. (2026) and Atkinson (2024), can be used to manage the volume of literature efficiently. Where such tools are used, the version, configuration, and human oversight protocol must be documented to preserve reproducibility of the review process, consistent with the requirements of PRISMA – AI. These five steps are intended to be applied sequentially, with the outcome of each step informing the scope and methodology of the next, thereby ensuring that synthesis decisions at every stage are grounded in an explicit, evidence-based assessment of the quality, transparency, and comparability of the underlying ML evidence.

Machine learning specific bias assessment and reporting standards are essential throughout the review process. PROBAST + AI (Moons et al., 2025) should guide bias assessment by addressing data leakage, algorithmic fairness, model interpretability, and updating procedures. Where subgroup data are available, reviewers should evaluate algorithmic bias across demographic and clinical populations (Obermeyer et al., 2019). Reporting should follow TRIPOD + AI (Collins et al., 2024) for primary studies and PRISMA – AI (Page et al., 2021) for SRs, ensuring transparency in model development, validation, and synthesis methods. Reproducibility assessment should document code availability, model weights, and training data, with federated learning approaches enabling validation without direct data sharing where necessary.

Figures 1A, B present a structured five-step framework integrating these considerations: assessing validation status and selecting synthesis methods, conducting ML-specific bias assessment, applying reporting standards, evaluating reproducibility, and considering AI-assisted automation with human oversight (van de Schoot et al., 2021; Daraio et al., 2026). This framework emphasizes methodological flexibility, synthesis strategies, bias assessment tools, and reproducibility checks should be tailored to the transparency and validation status of ML models under review. Rigid application of traditional methods may obscure rather than clarify the ML evidence base.

## 6 Future directions

### 6.1 Research gaps and future research priorities

Despite rapid advancements in machine learning for healthcare, several critical gaps remain in the evidence synthesis landscape. First, the scarcity of external validation studies limits the generalizability and reliability of pooled estimates. Second, fairness and equity assessments are rarely incorporated into systematic reviews, raising concerns about the ethical deployment of AI in clinical practice. Third, reproducibility remains constrained by limited access to datasets, algorithms, and source code. Fourth, heterogeneity in reporting standards and performance metrics continues to hinder meaningful comparisons across studies. Finally, methodological guidance for synthesizing continuously evolving and adaptive algorithms remains underdeveloped.

Addressing these gaps represents a priority for future research. Efforts should focus on developing robust methodological frameworks, establishing standardized performance metrics, and advancing approaches for evaluating dynamic and continuously learning models. Bridging these deficiencies will strengthen the reliability, transparency, and clinical applicability of systematic reviews of machine learning models in healthcare.

### 6.2 Recommendations

To address the challenges identified above, systematic review methodologies must evolve to ensure that evidence remains transparent, reproducible, and clinically relevant. The next phase of development should prioritize standardization, automation, and interdisciplinary collaboration.

There is an urgent need for consistent standards for reporting and bias assessment. Broad adoption of AI-specific frameworks, such as PROBAST + AI for bias evaluation (Moons et al., 2025), TRIPOD + AI for reporting (Collins et al., 2024), and PRISMA – AI for SR transparency (Page et al., 2021) should become mandatory in both primary and secondary ML research. Journals and funders can accelerate this by requiring adherence as a condition of publication or funding, such as the consolidated standards of reporting trials (CONSORT) (Liu et al., 2020) and PRISMA transformed clinical trial reporting.

Moreover, future SRs should adopt ongoing and automated workflows to keep pace with the rapidly expanding ML literature. Active learning and LLM-assisted screening can maintain review sensitivity while improving efficiency (van de Schoot et al., 2021; Daraio et al., 2026). Ongoing review platforms, integrated with PubMed and arXiv monitoring systems, can ensure that evidence syntheses remain current in fields where algorithms evolve monthly (Elliott et al., 2014). In addition, models should be evaluated across diverse populations and healthcare systems to confirm generalizability, with federated learning offering a practical path when direct data sharing is not feasible.

## 7 Limitations

This review is a narrative methodological evaluation and therefore has inherent limitations. It does not follow a pre-registered protocol or formal systematic search strategy, and no quantitative meta-analysis was performed. The analysis relies on previously published systematic reviews and illustrative case examples, which may be subject to reporting and publication bias, as well as heterogeneity in study quality. While representative studies were selected to reflect current practice, the possibility of selection bias cannot be fully excluded. Nevertheless, this approach is appropriate for examining cross-cutting methodological challenges and emerging solutions in a rapidly evolving field where formal synthesis is often infeasible. Notwithstanding these limitations, this review integrates recent high-impact studies and methodological advancements, ensuring that its conclusions reflect the current state of the art in ML evidence synthesis.

## 8 Conclusion

Systematic reviews of ML prediction models in healthcare face unique challenges arising from dynamic model behavior, data dependencies, algorithmic bias, and inconsistent reporting. Among these, the near-absence of external validation represents the most critical barrier to reliable evidence synthesis. Current evidence reveals substantial gaps: most models lack external validation, rarely assess fairness, and often do not provide code or data for reproducibility. Emerging solutions, including PROBAST + AI for bias assessment, PRISMA – AI for reporting, network meta-analysis for comparative evaluation, and FAIR-aligned open science practices, offer pathways to more rigorous and transparent evidence synthesis. Implementing these approaches requires multidisciplinary collaboration, methodological rigor, and ongoing adaptation to evolving ML technologies. By prioritizing external validation, fairness, and reproducibility, systematic reviews can bridge the gap between published performance and real-world clinical utility, supporting the safe and equitable integration of ML into healthcare.

## References

[B1] Alhumaidi N. H. Dermawan D. Kamaruzaman H. F. Alotaiq N. (2025). The use of machine learning for analyzing real-world data in disease prediction and management: systematic review. JMIR Med. Inform. 13:e68898. doi: 10.2196/68898 40537090 PMC12226786

[B2] Alsharid M. Guo X. Men Q. Saha P. Mishra D. Ahuja R. . (2025). On the public dissemination and open sourcing of ultrasound resources, datasets and deep learning models. NPJ. Digit. Med. 8:777. doi: 10.1038/s41746-025-02162-4 41286495 PMC12722232

[B3] Atkinson C. F. (2024). Cheap, quick, and rigorous: artificial intelligence and the systematic literature review. Soc. Sci. Comput. Rev. 42, 376–393. doi: 10.1177/08944393231196281

[B4] Beede E. Baylor E. Hersch F. Iurchenko A. Wilcox L. Ruamviboonsuk P. . (2020). “A human-centered evaluation of a deep learning system deployed in clinics for the detection of diabetic retinopathy,” in Proceedings of the 2020 CHI Conference on Human Factors in Computing Systems (Honolulu), 1–12. doi: 10.1145/3313831.3376718

[B5] Cacciamani G. E. Chu T. N. Sanford D. I. Abreu A. Duddalwar V. Oberai A. . (2023). PRISMA AI reporting guidelines for systematic reviews and meta-analyses on AI in healthcare. Nat. Med. 29, 14–15. doi: 10.1038/s41591-022-02139-w 36646804

[B6] Christodoulou E. Ma J. Collins G. S. Steyerberg E. W. Verbakel J. Y. Van Calster B. (2019). A systematic review shows no performance benefit of machine learning over logistic regression for clinical prediction models. J. Clin. Epidemiol. 110, 12–22. doi: 10.1016/j.jclinepi.2019.02.004 30763612

[B7] Collins G. S. Moons K. G. M. Dhiman P. Riley R. D. Beam A. L. Van Calster B. . (2024). TRIPOD+AI statement: updated guidance for reporting clinical prediction models that use regression or machine learning methods. BMJ 385:q902. doi: 10.1136/bmj-2023-078378 38626948 PMC11019967

[B8] Daraio C. Di Leo S. Ferazzoli F. (2026). Pitfalls, benefits, and comparative analysis of artificial intelligence ChatBots in the systematic review process. Int. Trans. Oper. Res. 33, 719–774. doi: 10.1111/itor.70054

[B9] Debray T. P. A. Moons K. G. M. Ahmed I. Koffijberg H. Riley R. D. (2013). A framework for developing, implementing, and evaluating clinical prediction models in an individual participant data meta-analysis. Stat. Med. 32, 3158–3180. doi: 10.1002/sim.5732 23307585

[B10] Elliott J. H. Turner T. Clavisi O. Thomas J. Higgins J. P. Mavergames C. . (2014). Living systematic reviews: an emerging opportunity to narrow the evidence-practice gap. PLoS Med. 11:e1001603. doi: 10.1371/journal.pmed.1001603 24558353 PMC3928029

[B11] Finlayson S. Subbaswamy A. Singh K. Bowers J. Kupke A. Zittrain J. . (2021). The clinician and dataset shift in artificial intelligence. N. Engl. J. Med. 3, 283–286. doi: 10.1056/NEJMc2104626 34260843 PMC8665481

[B12] Gao Y. Wang C. Shen J. Wang Z. Liu Y. Chai Y. . (2024). Systematic review and network meta-analysis of machine learning algorithms in sepsis prediction. Expert. Syst. Appl. 245:122982. doi: 10.1016/j.eswa.2023.122982

[B13] Gundersen O. E. Gil Y. Aha D. W. (2018). On reproducible AI: towards reproducible research, open science, and digital scholarship in AI publications. AI Mag. 3, 56–68. doi: 10.1609/aimag.v39i3.2816

[B14] Khattak M. U. Kunhimon S. Naseer M. Khan S. Khan F. S. (2024). UniMed-CLIP: towards a unified image-text pretraining paradigm for diverse medical imaging modalities. arXiv [preprint] arXiv:10372. doi: 10.48550/arXiv.2412.10372

[B15] Kolasa K. Admassu B. Hołownia-Voloskova M. Kedzior K. J. Poirrier J. E. Perni S. . (2024). Systematic reviews of machine learning in healthcare: a literature review. Expert. Rev. Pharmacoecon. Outcomes Res. 24, 63–115. doi: 10.1080/14737167.2023.2279107 37955147

[B16] Liu X. Cruz Rivera S. Moher D. Calvert M. J. Denniston A. K. Ashrafian H. . (2020). Reporting guidelines for clinical trial reports for interventions involving artificial intelligence: the CONSORT-AI extension. Nat. Med. 26, 1364–1374. doi: 10.1038/s41591-020-1034-x 32908283 PMC7598943

[B17] Moons K. G. M. Damen J. A. A. Kaul T. Hooft L. Navarro C. A. Dhiman P. . (2025). PROBAST+AI: an updated quality, risk of bias, and applicability assessment tool for prediction models using regression or artificial intelligence methods. BMJ 388:e082505. doi: 10.1136/bmj-2024-082505 40127903 PMC11931409

[B18] Nagendran M. Chen Y. Lovejoy C. A. Gordon A. C. Komorowski M. Harvey H. . (2020). Artificial intelligence versus clinicians: systematic review of design, reporting standards, and claims of deep learning studies. BMJ. 368:m689. doi: 10.1136/bmj.m689 32213531 PMC7190037

[B19] Norgeot B. Quer G. Beaulieu-Jones B. K. Torkamani A. Dias R. Gianfrancesco M. . (2020). Minimum information about clinical artificial intelligence modeling: the MI-CLAIM checklist. Nat. Med. 26, 1320–1324. doi: 10.1038/s41591-020-1041-y 32908275 PMC7538196

[B20] Obermeyer Z. Powers B. Vogeli C. Mullainathan S. (2019). Dissecting racial bias in an algorithm used to manage the health of populations. Am. Assoc. Adv. Sci. 366, 447–453. doi: 10.1126/science.aax2342 31649194

[B21] Offidani M. N. Roffet F. Galtier M. C. Massiris M. Delrieux C. (2025). An open-source clinical case dataset for medical image classification and multimodal AI applications. Data 10:123. doi: 10.3390/data10080123

[B22] Page M. J. Moher D. Bossuyt P. M. Boutron I. Hoffmann T. C. Mulrow C. D. . (2021). PRISMA 2020 explanation and elaboration: updated guidance and exemplars for reporting systematic reviews. BMJ. 372:n160. doi: 10.1136/bmj.n160 33781993 PMC8005925

[B23] Rajkomar A. Dean J. Kohane I. (2019). Machine learning in medicine. N. Engl. J. Med. 380, 1347–1358. doi: 10.1056/NEJMra1814259 30943338

[B24] Rakers M. M. van Buchem M. M. Kucenko S. de Hond A. Kant I. van Smeden M. . (2024). Availability of evidence for predictive machine learning algorithms in primary care: a systematic review. JAMA Netw. Open 7:e2432990. doi: 10.1001/jamanetworkopen.2024.32990 39264624 PMC11393722

[B25] Ramspek C. L. Jager K. J. Dekker F. W. Zoccali C. van Diepen M. (2021). External validation of prognostic models: what, why, how, when and where? Clin. Kidney J. 14, 49–58. doi: 10.1093/ckj/sfaa188 33564405 PMC7857818

[B26] Roberts M. Driggs D. Thorpe M. Gilbey J. Yeung M. Ursprung S. . (2021). Common pitfalls and recommendations for using machine learning to detect and prognosticate for COVID-19 using chest radiographs and CT scans. Nat. Mach. Intell. 3, 199–217. doi: 10.1038/s42256-021-00307-0

[B27] Rudin C. (2019). Stop explaining black box machine learning models for high stakes decisions and use interpretable models instead. Nat. Mach. Intell. 1, 206–215. doi: 10.1038/s42256-019-0048-x 35603010 PMC9122117

[B28] Sendak M. P. Gao M. Brajer N. Balu S. (2020). Presenting machine learning model information to clinical end users with model facts labels. NPJ Digit. Med. 3:41. doi: 10.1038/s41746-020-0253-3 32219182 PMC7090057

[B29] van de Schoot R. de Bruin J. Schram R. Zahedi P. De Boer J. Weijdema F. . (2021). An open source machine learning framework for efficient and transparent systematic reviews. Nat. Mach. Intell. 3, 125–133. doi: 10.1038/s42256-020-00287-7

[B30] Wallach J. D. Boyack K. W. Ioannidis J. P. A. (2018). Reproducible research practices, transparency, and open access data in the biomedical literature, 2015–2017. PLoS Biol. 16:e2006930. doi: 10.1371/journal.pbio.2006930 30457984 PMC6245499

[B31] Wong A. Otles E. Donnelly J. P. Krumm A. McCullough J. DeTroyer-Cooley O. . (2021). External validation of a widely implemented proprietary sepsis prediction model in hospitalized patients. JAMA Intern. Med. 181, 1065–1070. doi: 10.1001/jamainternmed.2021.2626 34152373 PMC8218233

[B32] Wynants L. Van Calster B. Collins G. S. Riley R. D. Heinze G. Schuit E. . (2020). Prediction models for diagnosis and prognosis of covid-19: systematic review and critical appraisal. BMJ 369:m1328. doi: 10.1136/bmj.m1328 32265220 PMC7222643

